# Compressional Optical Coherence Elastography of the Cornea

**DOI:** 10.3390/photonics8040111

**Published:** 2021-04-07

**Authors:** Manmohan Singh, Achuth Nair, Salavat R. Aglyamov, Kirill V. Larin

**Affiliations:** 1 Department of Biomedical Engineering, University of Houston, 3517 Cullen Blvd., Room 2027, Houston, TX 77204, USA; 2 Department of Mechanical Engineering, University of Houston, 4726 Calhoun Rd., Room N207, Houston, TX 77204, USA; 3 Molecular Physiology and Biophysics, Baylor College of Medicine, One Baylor Plaza, BCM335, Houston, TX 77030, USA

**Keywords:** optical coherence elastography, biomechanics, stiffness, elasticity, cornea

## Abstract

Assessing the biomechanical properties of the cornea is crucial for detecting the onset and progression of eye diseases. In this work, we demonstrate the application of compression-based optical coherence elastography (OCE) to measure the biomechanical properties of the cornea under various conditions, including validation in an in situ rabbit model and a demonstration of feasibility for in vivo measurements. Our results show a stark increase in the stiffness of the corneas as IOP was increased. Moreover, UV-A/riboflavin corneal collagen crosslinking (CXL) also dramatically increased the stiffness of the corneas. The results were consistent across 4 different scenarios (whole CXL in situ, partial CXL in situ, whole CXL in vivo, and partial CXL in vivo), emphasizing the reliability of compression OCE to measure corneal biomechanical properties and its potential for clinical applications.

## 1. Introduction

The cornea provides approximately 2/3 of the total refracting power of the eye [1]. Its innate biomechanical properties give rise to its shape and, ultimately, its function. Various diseases such as keratoconus [2] can alter corneal biomechanical properties, and it has been postulated that biomechanical changes in the cornea precede structural changes. Therefore, biomechanical imaging of the cornea could enable earlier detection of disease, minimizing loss of visual acuity. Moreover, surgical interventions, such as laser assisted in situ keratomileusis (LASIK) and UV-A/riboflavin corneal collagen crosslinking (CXL) can significantly alter corneal biomechanical properties [3]. Therefore, various techniques have been developed to assess the biomechanical properties of the cornea, including established techniques such as magnetic resonance elastography [4] and ultrasound elastography [5,6]. Although these techniques have been immensely useful for clinical applications in oncology [7] and hepatology [8], they have limited use in the cornea due to their spatial resolution and requirement of physical access to the cornea. Clinically available instruments such as the Ocular Response Analyzer (Reichert Tech., Depew, NY, USA) and Corvis (Oculus, Inc., Arlington, WA, USA) have shown promise for detecting corneal diseases from their biomechanical measurements. However, there is conflicting literature on their efficacy for detecting disease and the outcomes of therapies [9–11], and their biomechanical measurements are confounded by numerous parameters such as corneal thickness, curvature, and intraocular pressure (IOP) [12]. Moreover, the large amplitude displacements limit their ability to map corneal biomechanical properties and are prone to non-linear biomechanical responses that make quantifications of corneal elasticity difficult. Brillouin microscopy is emerging as a tool for assessing corneal biomechanics, but translating Brillouin measurements into quantitative material parameters is an open question [13], and long imaging times limit its applicability for live imaging [14]. Therefore, there is a rising interest in utilizing faster and higher resolution techniques for imaging corneal biomechanical properties, particularly with optical coherence tomography [15] based elastography [16], which is termed optical coherence elastography (OCE) [17–19].

In contrast to wave-based OCE techniques, static/quasi-static OCE techniques show promise for high-resolution elasticity mapping, albeit with contact to the tissue [20]. Wave-based techniques rely on high-frequency content to approach the mechanical resolutions available in static techniques, but high frequency waves attenuate faster and limit the mechanical field of view [17]. Moreover, improved mechanical contrast and high frequency excitation techniques can also require contact with the tissue [21]. Compression based OCE detected spatial variations in the elasticity of the cornea [22,23] and measured corneal stiffness in situ under various conditions [24,25]. However, these studies rely on speckle tracking, which suffers from decorrelation artifacts and has limited sensitivity to motion. Moreover, the induced displacements were large (approaching 1 mm), which caused bulk motion and non-linear tissue responses resulting in difficult quantitation of material properties (e.g., Young’s modulus). In contrast, phase-sensitive approaches have been robustly developed for high-resolution and high-sensitivity elasticity mapping [20], enabling the use of minimal force that limits or even eliminates bulk motion and non-linear tissue responses. Phase-sensitive compression OCE [20] has been utilized to monitor thermal effects on corneas for laser reshaping [26,27]. However, there have been no studies elucidating the changes in corneal stiffness due to changes in IOP and/or CXL. In this work, we demonstrate that phase-sensitive compression OCE can detect spatial variations in the cornea and changes in corneal elasticity after CXL. Experiments were performed on in situ rabbit corneas under various conditions for validation, and then the feasibility of the technique for in vivo measurements was demonstrated in a rabbit model.

## 2. Materials and Methods

### 2.1. Cornea Samples

Fresh whole rabbit eye globes (N = 4, Pel-Freez LLC, Rogers, AR, USA) were shipped overnight on ice. The eyes were visually examined for any damage, and only undamaged sampled were utilized. The eye globes were placed in a custom holder and cannulated during the in situ experiments for artificial IOP control by a previously described closed-loop system [28]. Corneas (N = 3) were imaged at baseline IOPs of 10, 15, 20, 25, and 30 mmHg, and then CXL was performed. The CXL procedure mimicked the clinically established “Dresden” protocol [29]. Briefly, the epithelium was removed with a blunt spatula, and a 0.1% riboflavin-5-phosphate in 20% dextran solution was added topically every 5 min for 30 min. The corneas were then irradiated with 365 nm light at 3 mW/cm^2^ for 30 min, during which the riboflavin solution was also applied every 5 min. Following CXL, the OCE measurements were repeated. To assess the ability of the system to measure spatial differences in corneal elasticity, a custom CXL procedure was also performed on one sample where only half of the cornea was irradiated.

In vivo imaging was performed on anesthetized adult Dutch-belted rabbits (N = 2). In the first animal, OCE imaging was performed before and after the traditional “Dresden” CXL procedure. In the second animal, OCE imaging was performed after a custom CXL procedure as described earlier, where only half of the cornea was irradiated. Anesthesia was induced with an intramuscular dose of ketamine (40 mg/kg) and xylazine (5 mg/kg). Subsequent maintenance doses (20 mg/kg of ketamine) were administered as needed based on physiological responses (toe pinch, corneal response, respiration, and heart rate). All animal procedures were approved by the University of Houston Institutional Animal Care and Use Committee and performed by trained veterinary personnel.

### 2.2. OCE Imaging

**Figure 1.**
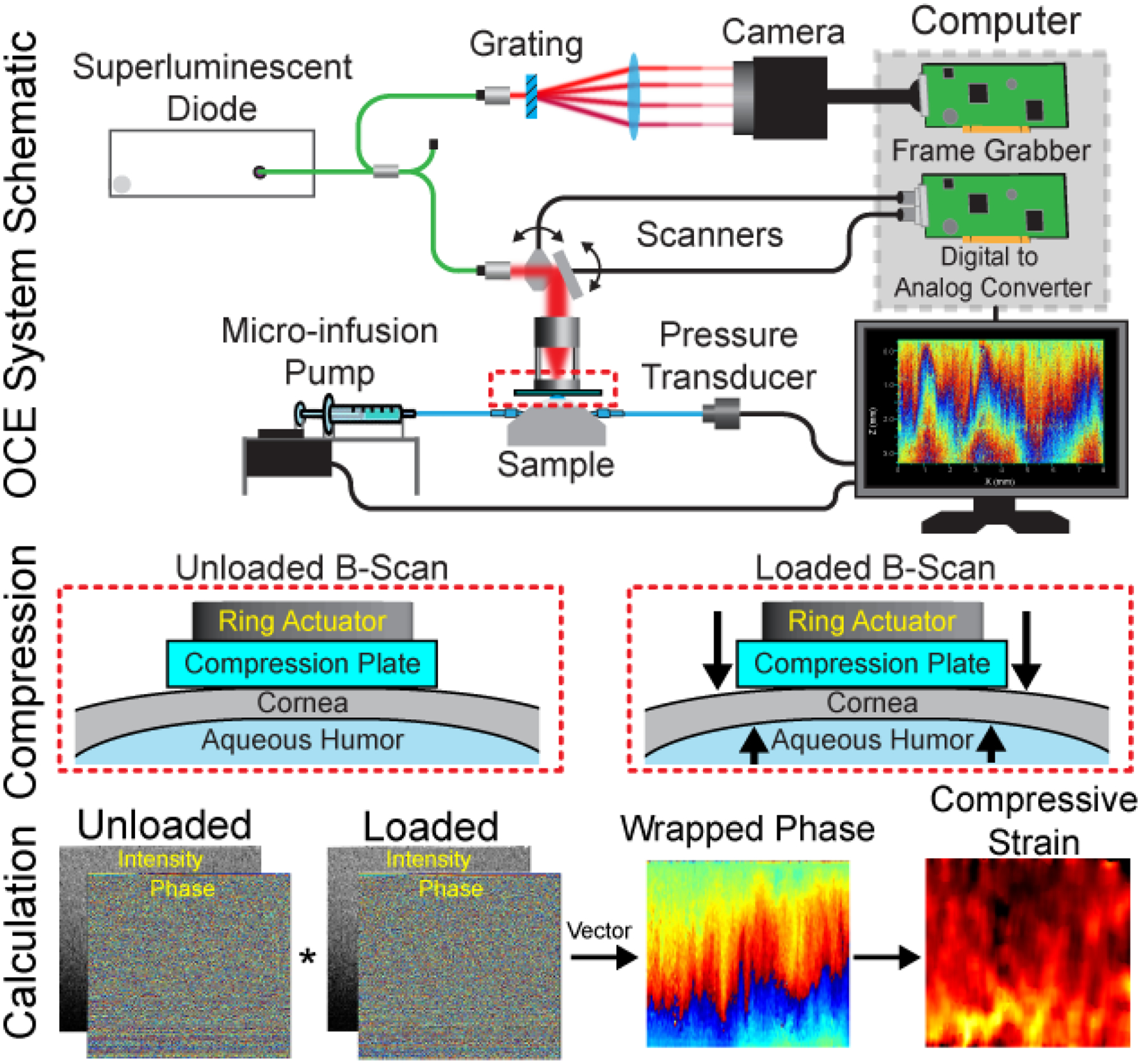
Optical coherence elastography (OCE) imaging and data processing paradigm. (**top**) Schematic of the OCE system during in situ imaging of the rabbit corneas. (**middle**) Compression OCE imaging paradigm. (**bottom**) Strain mapping calculation steps. * denotes complex conjugate multiplication.

OCE imaging was performed with a home-built phase-sensitive spectral domain OCT system, which has been described previously [30]. A schematic of the OCE setup during the in situ corneal measurements is shown at the top of Figure 1. The setup was based on a Michelson interferometer but was operated in common path mode to improve the sensitivity of the system to displacements and remove the influence of environmental noise [31–33]. Broadband light (central wavelength: 840 nm, bandwidth: 49 nm) from a superluminescent diode (Broadlighter S840-I-B-20, Superlum, Cobh Cross, Ireland) was split by a 50/50 fiber coupler to the sample arm and spectrometer. The axial resolution was ~8 μm in air with a lateral resolution of ~9 μm, also in air. To compress the cornea, a ring piezo-electric actuator (HPSt 150/14–10/12 Piezomechanik GmbH, Munich, Germany) was attached to the sample arm on one end and a glass plate on the other end. The actuator was synchronized with the OCE frame trigger such that one image was taken while the cornea was unloaded, and the next image was taken when the sample was loaded, as shown in the compression OCE methodology in the middle of Figure 1. A total of 20 images were taken (10 unloaded and 10 loaded) during OCE imaging. During the in situ measurements, the IOP was also recorded synchronously. Imaging was performed with 2000 A-lines per B-scan over ~8 mm with the camera line rate at 25 kHz (resulting in a frame time of 80 ms).

During in vivo imaging, the animal heart rate was recorded and synchronized with the OCE measurements to eliminate the influence of the ocular pulse on the measurements [30]. Due to the presence of motion in the live animals, a reduced scan area and higher frame rate were utilized to minimize the effects of bulk motion and any other extraneous noise. The OCE imaging was performed over ~2.6 mm during in vivo imaging with 1000 A-lines per B-scan and a camera line rate of 62.5 kHz (resulting in a frame time of 16 ms).

In all cases, the actuator was driven with identical amplitude signals to minimize any inconsistencies from loading. In the air, the unloaded actuator displacement was ~8 μm. The actuator loading rates were identical to the frame rate of the system. The low frequency excitation (tens of Hz) ensured there was a minimal effect of viscosity on the measurements [34]. The vector method was utilized to obtain the phase difference between successive B-scans [35], as shown at the bottom of Figure 1. An isometric kernel size of ~20 μm was utilized during processing. Although 10 B-scan pairs were imaged (a total of 20 OCT B-scans), 19 B-scan pairs were utilized for the strain calculations. We utilized the phase difference between each frame and flipped the sign of the phase difference between every other phase difference image to increase the total number of phase difference images from 10 to 19. The phase differences, *φ*, were unwrapped and then converted to displacement, *d*, by

(1) $$d=\frac{\varphi \lambda_{0}}{4\pi n},$$

assuming a refractive index, *n*, of 1.376 [36], and λ_0_ was the central wavelength of the OCT system. The strain was calculated by least squares regression method [37], where a quasi-2D processing method was utilized. The displacement was averaged laterally over ~100 μm, and the averaged displacement was then fitted over ~75 μm axially. These values were determined empirically to maximize the tradeoff between spatial resolution and SNR [38]. The compressive strain was then mapped for each sample. In this work, positive displacement was upwards to the compression plate. Generally, the strain is considered negative during compression, but for simplicity, we use the term compressive strain to indicate compression upwards towards the compression plate (i.e., squeezing of the sample). To minimize the effects of friction [37], a small drop of mineral oil was placed at the cornea apex, and the compressive plate was brought into contact with the cornea apex. The scan head was then lowered such that the cornea was applanated in the entire field of view. Regions at the edges where there was poor contact were not used for further analysis.

The strains were utilized to assess the stiffness of the cornea. However, in the in situ samples, the IOP was measured during the experiments, and there was a noticeable change in IOP between the unloaded and loaded phases of imaging. Hence, a pseudo-elasticity, *S*, was further calculated by

(2) $$S=\frac{\Delta \text{IOP}}{\varepsilon_{\text{comp}}}$$

where ΔIOP was the change in IOP between the unloaded and loaded states of the cornea, and *ε*_*comp*_ was the compressive strain.

### 2.3. Statistical Analyses

Statistical testing was performed by a Kruskal–Wallis ANOVA for the analyses as a function of IOP and Wilcoxon signed-rank test for the comparison before and after CXL, unless otherwise noted. A one-sided Wilcoxon signed-rank test was also used to determine if there was a significant difference between regions and treatment conditions under the assumption that the anterior region is stiffer than the posterior and that CXL stiffens the corneal tissue to increase the power of the test.

## 3. Results

### 3.1. In Situ Rabbit Corneas

**Figure 2.**
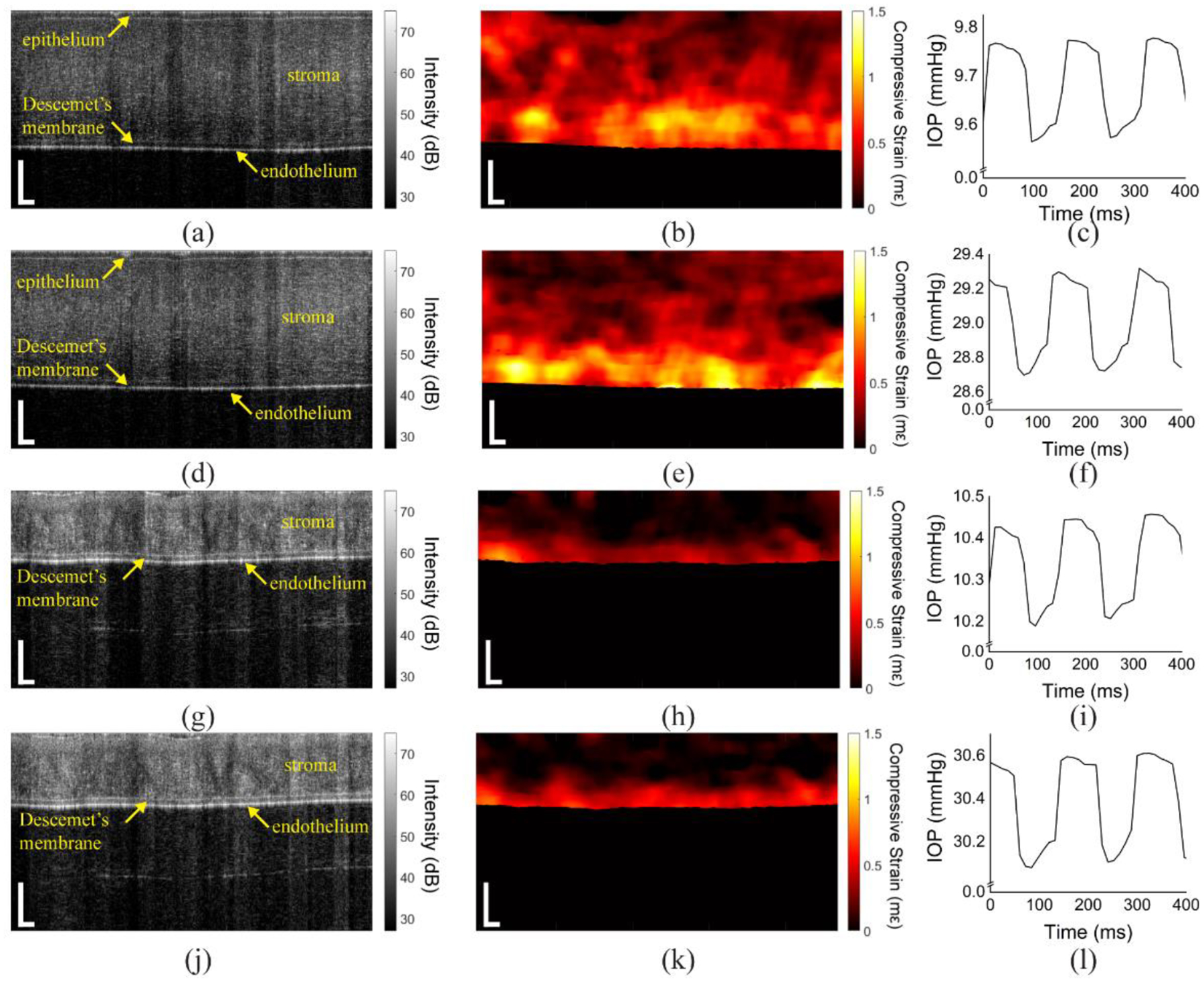
(left column) OCT images, (middle column) strain maps, and intraocular pressure (IOP) of typical in situ rabbit corneas. (**a**–**c**) A typical untreated sample at a baseline of ~10 mmHg and the same sample at (**d**–**f**) a baseline of ~29 mmHg. (**g**–**i**) The same sample after corneal collagen crosslinking (CXL) at a baseline of ~10 mmHg and at (**j**–**l**) a baseline of ~30 mmHg. Scale bars are 250 μm.

Results from a typical sample are shown in Figure 2 where the left column is OCT images, the middle column is strain maps, and the right column is the IOP. Figure 2a–c is from a virgin sample at a baseline IOP of ~10 mmHg, and Figure 2d–f is from the same virgin sample at a baseline IOP of ~30 mmHg. Figure 2g–i is from the same cornea after CXL at a baseline IOP of ~10 mmHg and Figure 2j–l ~30 mmHg. The compression plate is located at the top of the image. There was a noticeable change in thickness after CXL, which was expected due to the dextran solution. The changes in strain due to the baseline IOP are not directly evident, but there is a very clear reduction in strain after CXL, indicating stiffening of the cornea after CXL.

**Figure 3.**
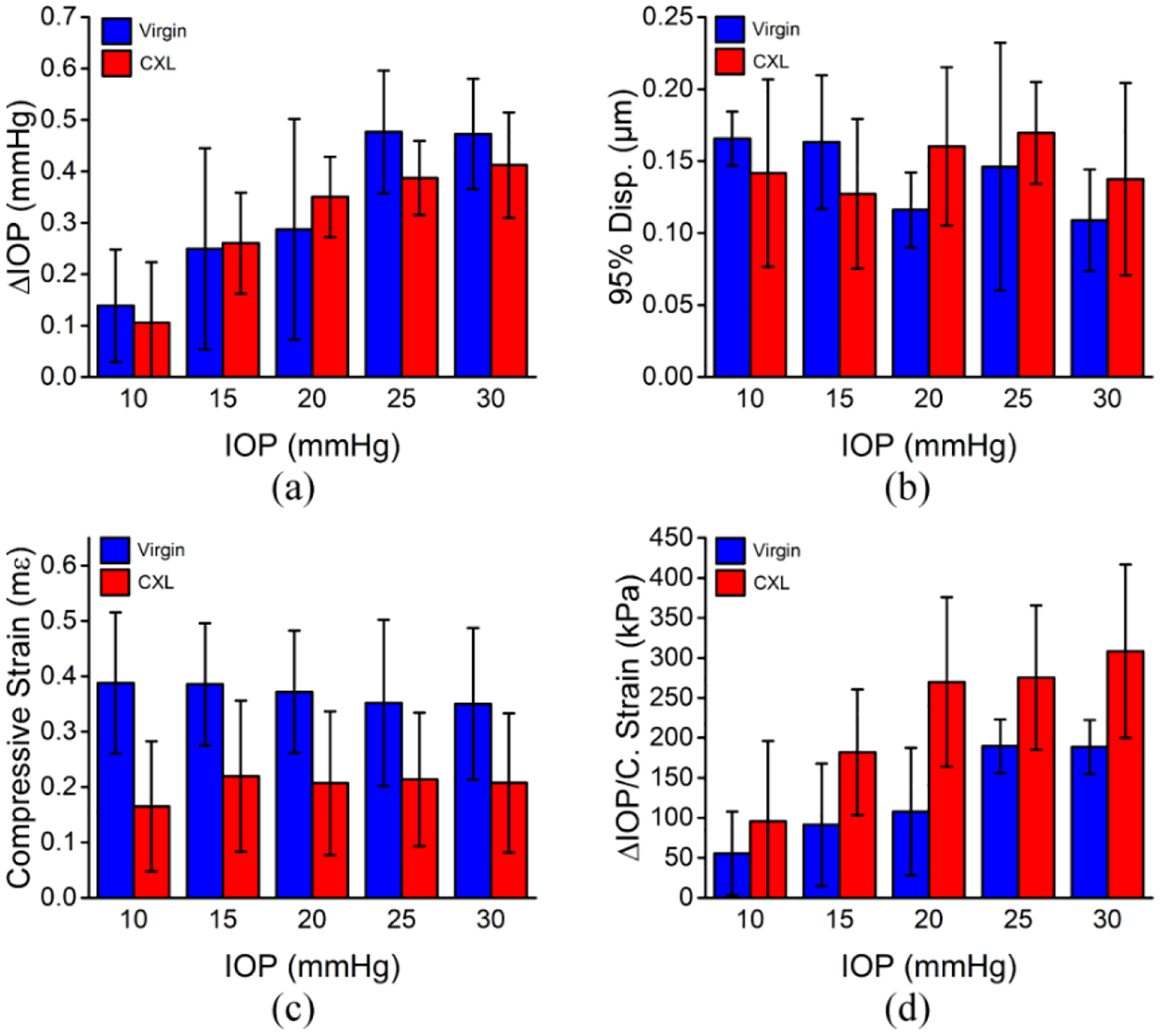
Results of the in situ rabbit corneas (N = 3) as a function of the baseline IOP and before and after CXL. (**a**) The difference in the IOP between the unloaded and loaded states, (**b**) 95th percentile of displacement, (**c**) strain, and (**d**) stiffness as quantified as the ΔIOP/strain as a function of the baseline IOP and before (virgin) and after CXL. The data are presented as the inter-sample means, and the error bars are the inter-sample standard deviation.

Figure 3 plots the results from all 3 in situ samples. Figure 3a shows the difference in IOP between the unloaded and loaded states for the samples before and after CXL and as a function of baseline IOP. The data are reported as the inter-sample mean ± inter-sample standard deviation unless noted otherwise. The ΔIOP for the virgin corneas at a baseline of 10 mmHg was 0.14 ± 0.11 mmHg, which increased to 0.47 ± 0.11 mmHg at a baseline IOP of 30 mmHg. After CXL, the ΔIOP at 10 mmHg was 0.11 ± 0.11 mmHg, which increased to 0.41 ± 0.10 mmHg at a baseline IOP of 30 mmHg. There was a significant difference in the ΔIOP as a function of baseline IOP (P = 0.003), but there was no significant difference in the ΔIOP before and after CXL (P = 0.761).

Due to the presence of noise and outliers, we quantified the 95th percentile of the displacements from every pixel inside the corneas instead of the maximum displacement, which is plotted in Figure 3b. For the virgin corneas, the displacement was 0.17 ± 0.02 μm at a baseline IOP of 10 mmHg, which decreased to 0.11 ± 0.14 μm at a baseline IOP of 30 mmHg. After CXL, the displacement at a baseline IOP of 10 mmHg was 0.14 ± 0.07 μm, which was also 0.14 ± 0.07 μm at a 30 mmHg baseline. Overall, there was no significant trend in the 95th percentile of displacement as a function of baseline IOP (P = 0.717) or CXL treatment (P = 0.335).

The strain similarly showed no trend as a function of baseline IOP (P = 0.989). At 10 mmHg baseline IOP for the virgin corneas, the strain was 0.39 ± 0.13 mε, which decreased slightly to 0.35 ± 0.14 mε at a baseline IOP of 30 mmHg. In contrast, there was a significant difference in the strain as a function of CXL treatment (P < 0.001), where the strain was 0.17 ± 0.11 mε and 0.21 ± 0.13 mε at baseline IOPs of 10 and 30 mmHg, respectively.

The stiffness as measured by ΔIOP/strain, however, was significant as a function of the baseline IOP (P = 0.037) and CXL treatment (P < 0.001). The stiffness of virgin corneas at a baseline IOP of 10 mmHg was 56 ± 52 kPa, which increased to 189 ± 34 kPa at 30 mmHg. After CXL at baseline IOPs of 10 and 30 mmHg, the corneal stiffness was 96 ± 100 kPa and 308 ± 108 kPa, respectively. There was an overall increase in the stiffness of the corneas of ~85% after CXL.

**Figure 4.**
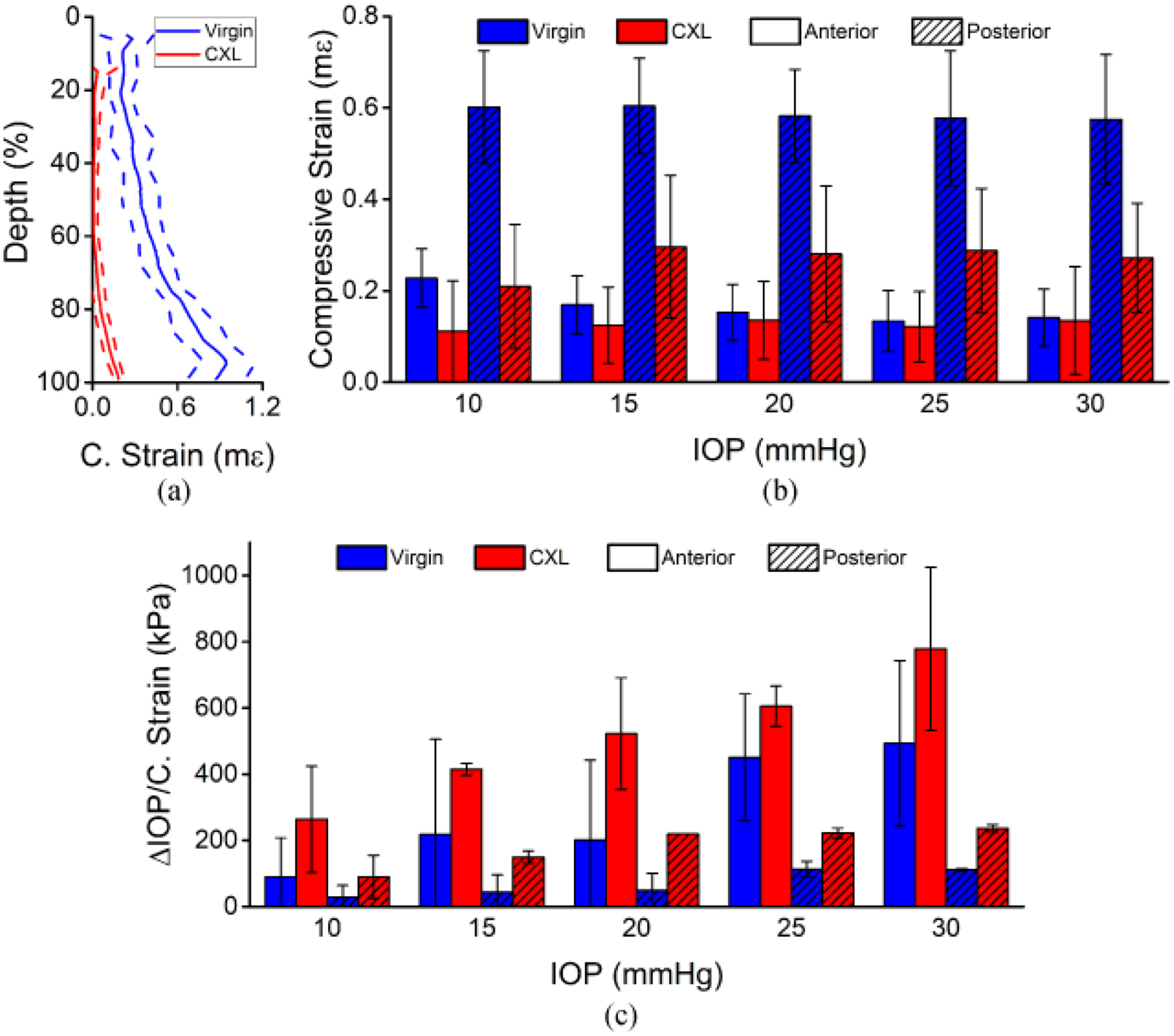
Depth-wise analysis of the in situ rabbit corneas (N = 3). (**a**) Example depth-wise strain averaged across the entire frame for a typical sample before and after CXL at 10 mmHg IOP. The solid line is the average, and the dashed lines show one standard deviation of error. The data was normalized to the full thickness. (**b**) Average compressive strain and (**c**) stiffness quantified by Equation (2) for each region shown as the intra-region mean ± standard deviation.

Figure 4 shows the results of the depth-wise strain analysis. As shown in Figure 4a, there was a clear difference between the anterior and posterior regions before and after CXL in the data from a typical sample. Figure 4b plots the summary of the strain from all 3 in situ samples separated by each region, where the anterior and posterior regions are the respective halves of each sample. The strain was 194% less in the anterior of the cornea as compared to posterior, which was very significant (P < 0.001), but the dependence on the strain as a function of the baseline IOP was not significant for either the anterior (P = 0.837) or posterior regions (P = 0.993). CXL decreased the strain in the anterior half of the corneas by ~55%, which was slightly significant (P = 0.042), and the strain decreased by ~62% in the posterior half of the corneas after CXL, which was significant (P = 0.003). The stiffness as quantified by Equation (2) was significant as a function of the baseline IOP in the anterior (P = 0.018) and posterior regions (P = 0.026). The stiffness of the posterior half of the corneas was ~69% softer than the anterior half, which was very significant (P < 0.001). The stiffness of the anterior half of the corneas increased by ~63% after CXL, which was significant (P = 0.038). CXL increased the stiffness of the posterior half of the corneas by 177%, which was also significant (P = 0.005).

**Figure 5.**
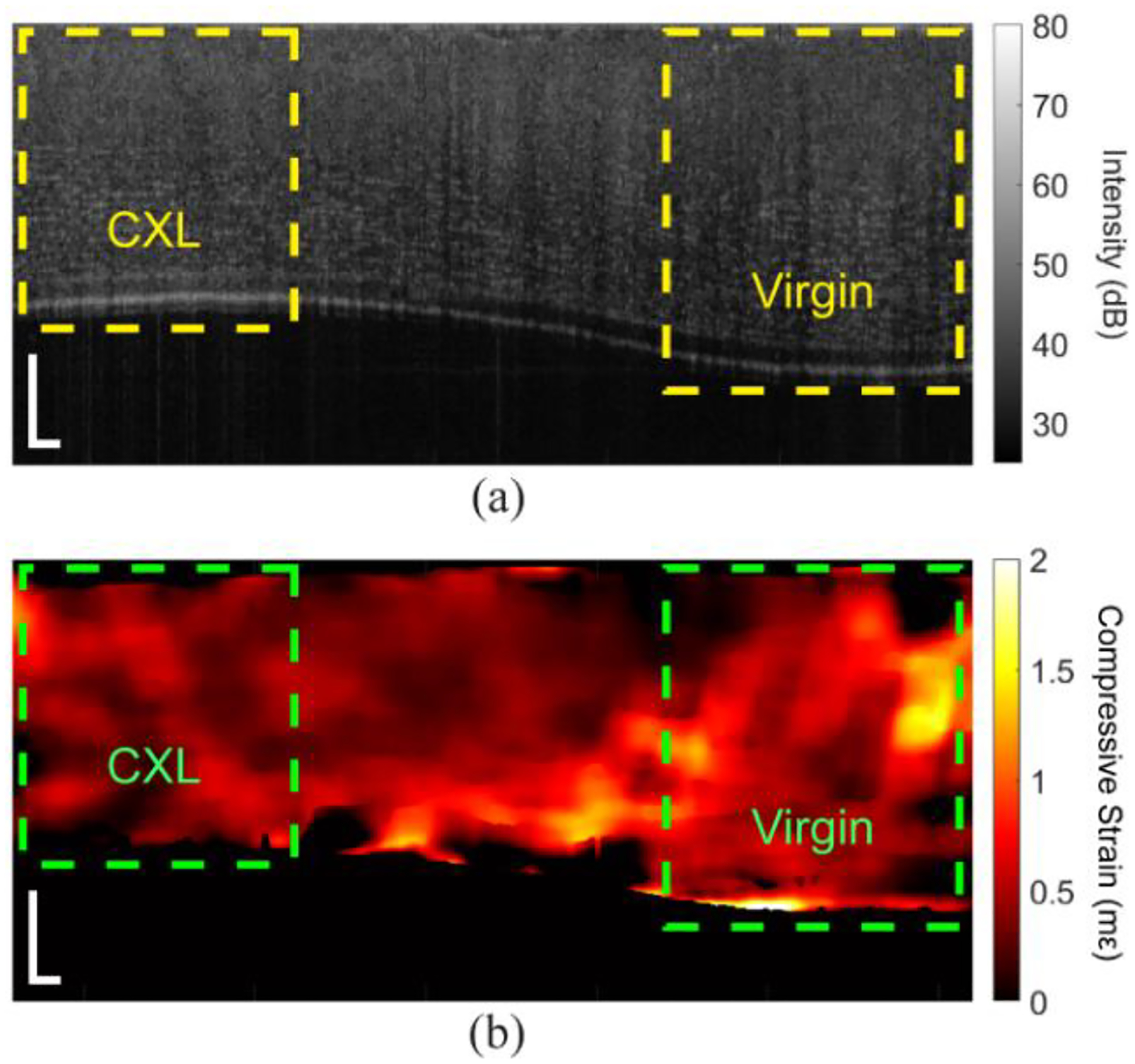
OCT and OCE images of the half-CXL in situ rabbit cornea. (**a**) OCT image and (**b**) strain map at 10 mmHg IOP. The left side of the cornea was CXL, where the right side was not irradiated with the UV light. The dashed boxes show the regions utilized for analysis. Scale bars are 250 μm.

In addition to the whole-CXL samples, partial CXL was performed on one sample, where only half of the sample was irradiated with the UV light. Figure 5 shows the OCT image and strain map of the half-CXL cornea at 10 mmHg baseline IOP. The dashed regions show the areas utilized for analysis. There is a clear difference in thickness of the samples that was used to delineate the different regions of the cornea.

**Figure 6.**
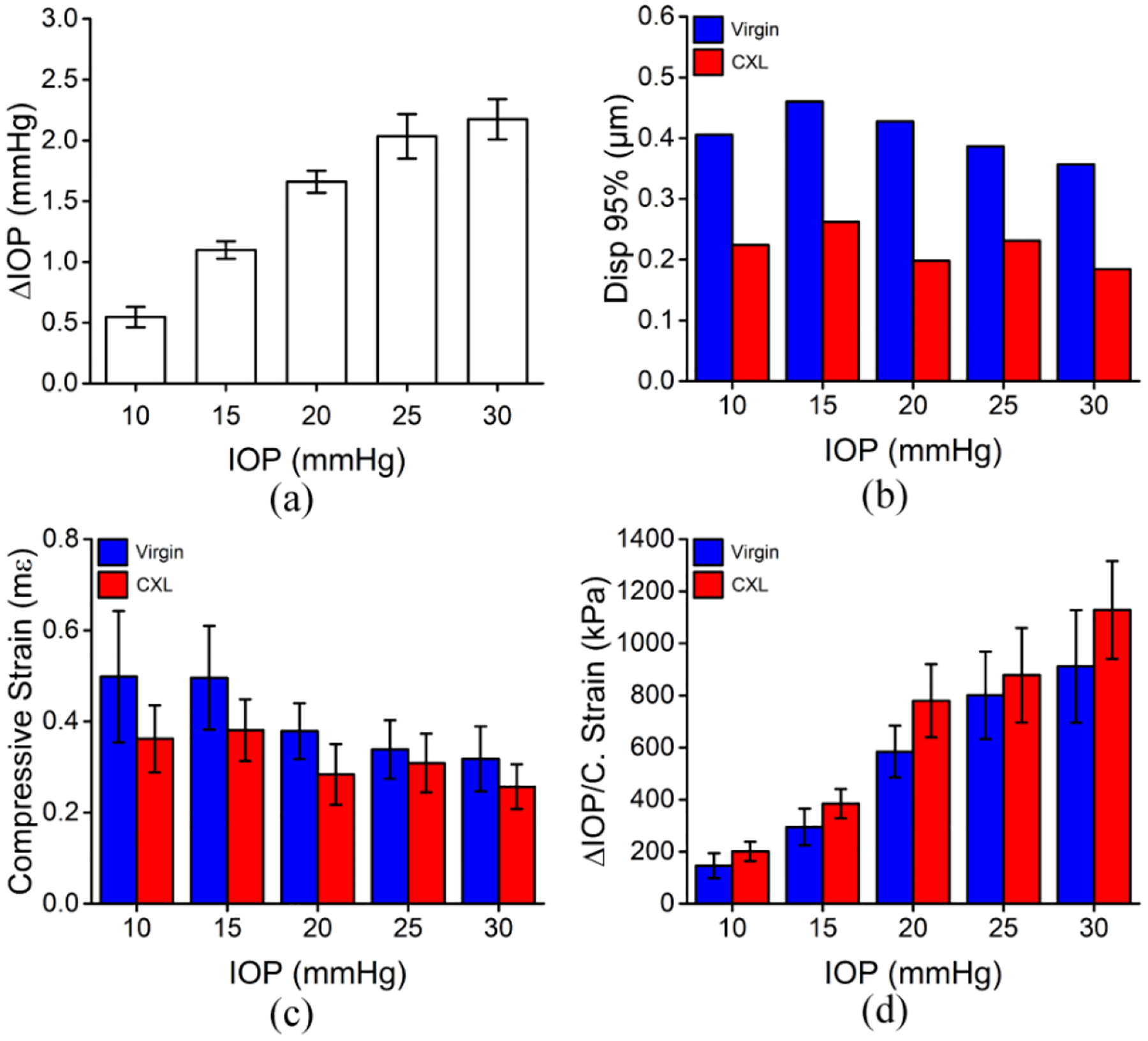
Results of the half-CXL in situ rabbit cornea sample (N = 1). (**a**) The difference in IOP between the unloaded and loaded states, (**b**) 95th percentile of the displacement, (**c**) strain, and (**d**) stiffness as quantified by ΔIOP/strain as a function of baseline IOP. The data is plotted as the inter-cycle mean ± standard deviation in (**a**) and intra-region mean ± standard deviation in (**c**,**d**).

For the half-CXL in situ samples, the data are presented as the intra-region means ± standard deviation. Statistical tests were performed as mentioned earlier unless otherwise noted. Figure 6 plots the results from the half-CXL sample. The mean peak-to-peak IOP is plotted in Figure 6a, which increased from 0.55 ± 0.08 mmHg at a baseline IOP of 10 mmHg to 2.17 ± 0.17 mmHg at a 30 mmHg baseline IOP. The ΔIOP was much greater as compared to the samples shown in Figure 3, which may have been due to differences in the contact between the corneas and the compressive plate and the fact that this sample was half-CXL with a very heterogeneous thickness. Figure 6b shows that the 95th percentile of the displacement for the virgin region was 0.41 μm and 0.36 μm at baseline IOPs of 10 mmHg and 30 mmHg, which were 0.22 and 0.18 in the CXL regions, respectively. The 95th percentile of the displacement was marginally significant between the two regions (P = 0.030). As plotted in Figure 6c, the average compressive strain of the virgin region at the 10 mmHg baseline was 0.60 ± 0.34 mε, which decreased to 0.49 ± 0.28 mε at a 30 mmHg baseline. In the CXL region, the average compressive strain was 0.37 ± 0.13 mε and 0.27 ± 0.10 mε at baseline IOPs of 10 and 30 mmHg, respectively. The average compressive strain was significant between the virgin and CXL parts of the cornea (P = 0.030). Figure 6d shows that the stiffness of the virgin region was 122 ± 73 kPa at 10 mmHg baseline, which increased to 590 ± 341 kPa at a baseline of 30 mmHg, and the stiffness of the CXL region increased from 197 ± 47.4 kPa to 1075 ± 230 kPa from a baseline IOP of 10 to 30 mmHg. There was a significant increase in stiffness after CXL (P = 0.030).

**Figure 7.**
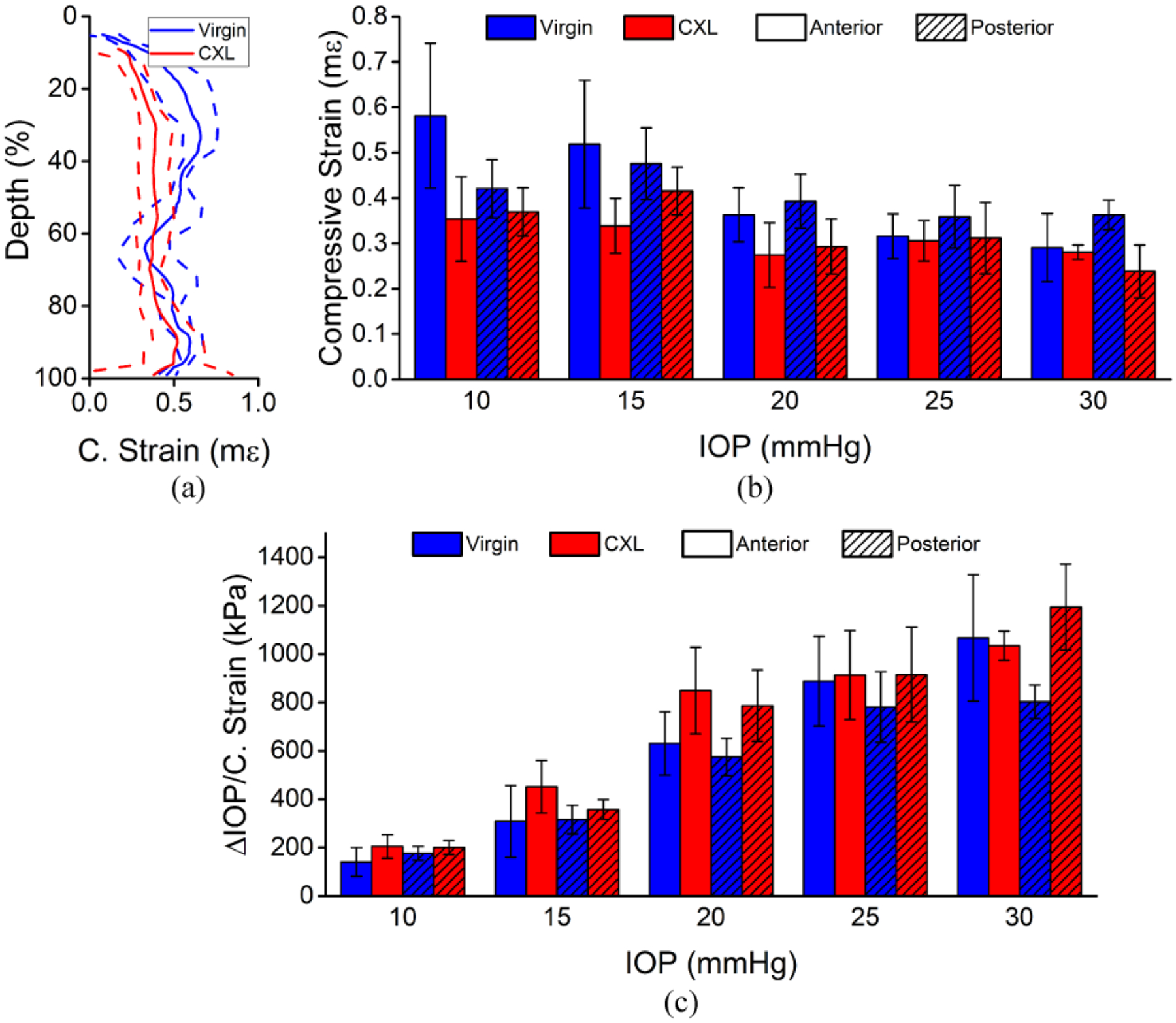
Depth-wise analysis of the half-CXL in situ rabbit cornea (N = 1). (**a**) Example depth-wise compressive strain averaged across the corresponding regions marked in Figure 5 at 15 mmHg IOP. The solid line is the average, and the dashed lines show one standard deviation of error. The data was normalized to the full thickness of the sample. (**b**) Average compressive strain and (**c**) stiffness quantified by Equation (2) for each region shown as the intra-region mean ± standard deviation.

Figure 7 shows the depth-resolved analysis of the half-CXL in situ rabbit cornea. In Figure 7a, the laterally averaged depth-wise compressive strains corresponding to the regions marked in Figure 5 are plotted. The corresponding summary of the compressive strain for each region is shown in Figure 7b. There was no significant difference in the compressive strain in the anterior versus posterior of the half-CXL sample (P = 0.730). The compressive strain in the anterior region was not significantly affected by the baseline IOP (P = 0.264), which was the same for the posterior region (P = 0.199). There was a marginally significant decrease in the compressive strain after CXL for the anterior region (P = 0.030) and posterior region (P = 0.030). Figure 7b plots the regional analysis of the stiffness as quantified by Equation (2). Although there is a clear trend in the stiffness as a function of the baseline IOP, it was not significant for the anterior region (P = 0.068) or the posterior region (P = 0.093). Similarly, there was no statistical difference between the stiffness of the anterior and posterior regions (P = 0.154). The anterior half of the virgin region was not significantly stiffened by CXL (P = 0.089), but the posterior half was significantly stiffened (P = 0.030).

### 3.2. In Vivo Rabbit Corneas

**Figure 8.**
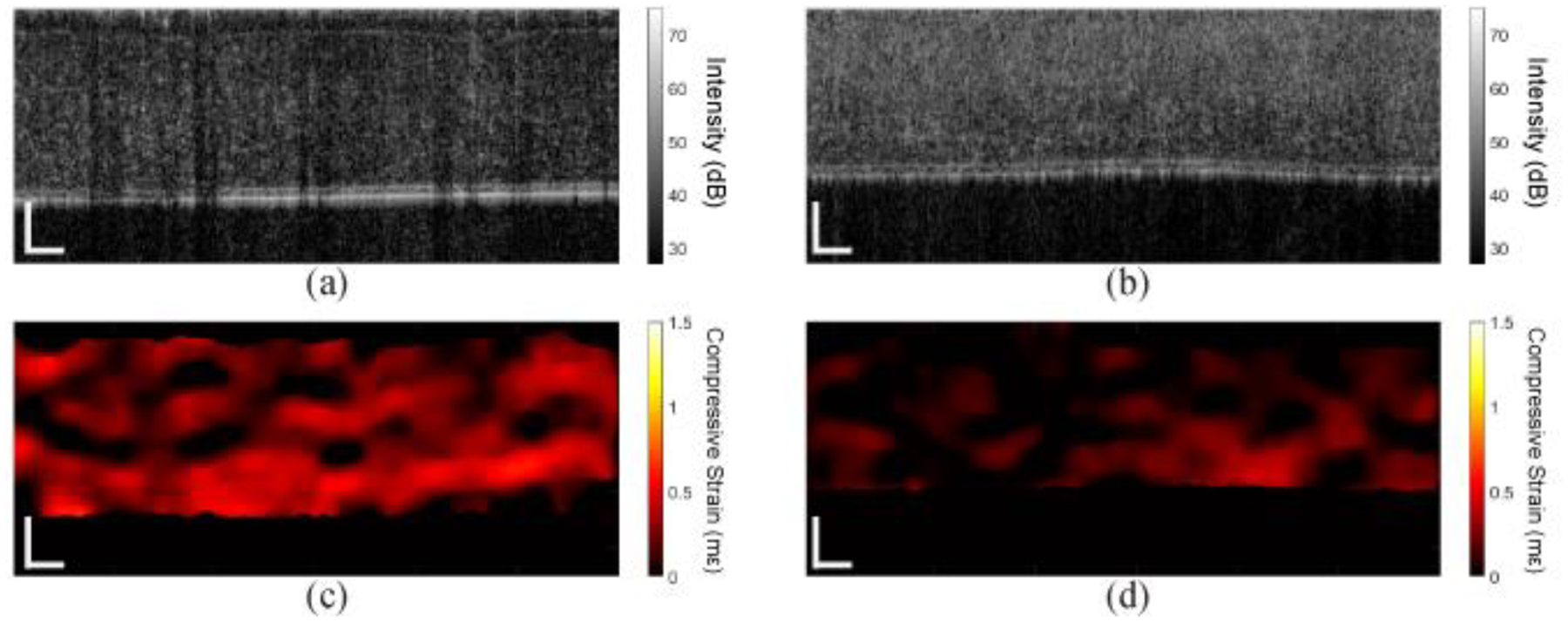
OCT and OCE images of the in vivo rabbit cornea. OCT images (**a**) before and (**b**) after CXL. Compressive strain maps of the cornea (**c**) before and (**d**) after CXL. Scale bars are 100 μm.

Results from the in vivo rabbit cornea are shown in Figure 8. Figure 8a shows the OCT image of the virgin cornea, and Figure 8b shows the OCT image of the same cornea after CXL. The compressive strain maps of the cornea before and after CXL are shown in Figure 8c,d, respectively. In contrast to the in situ sample, the change in thickness is not quite as dramatic. However, the clear decrease in strain shows the stiffening effects of CXL.

**Figure 9.**
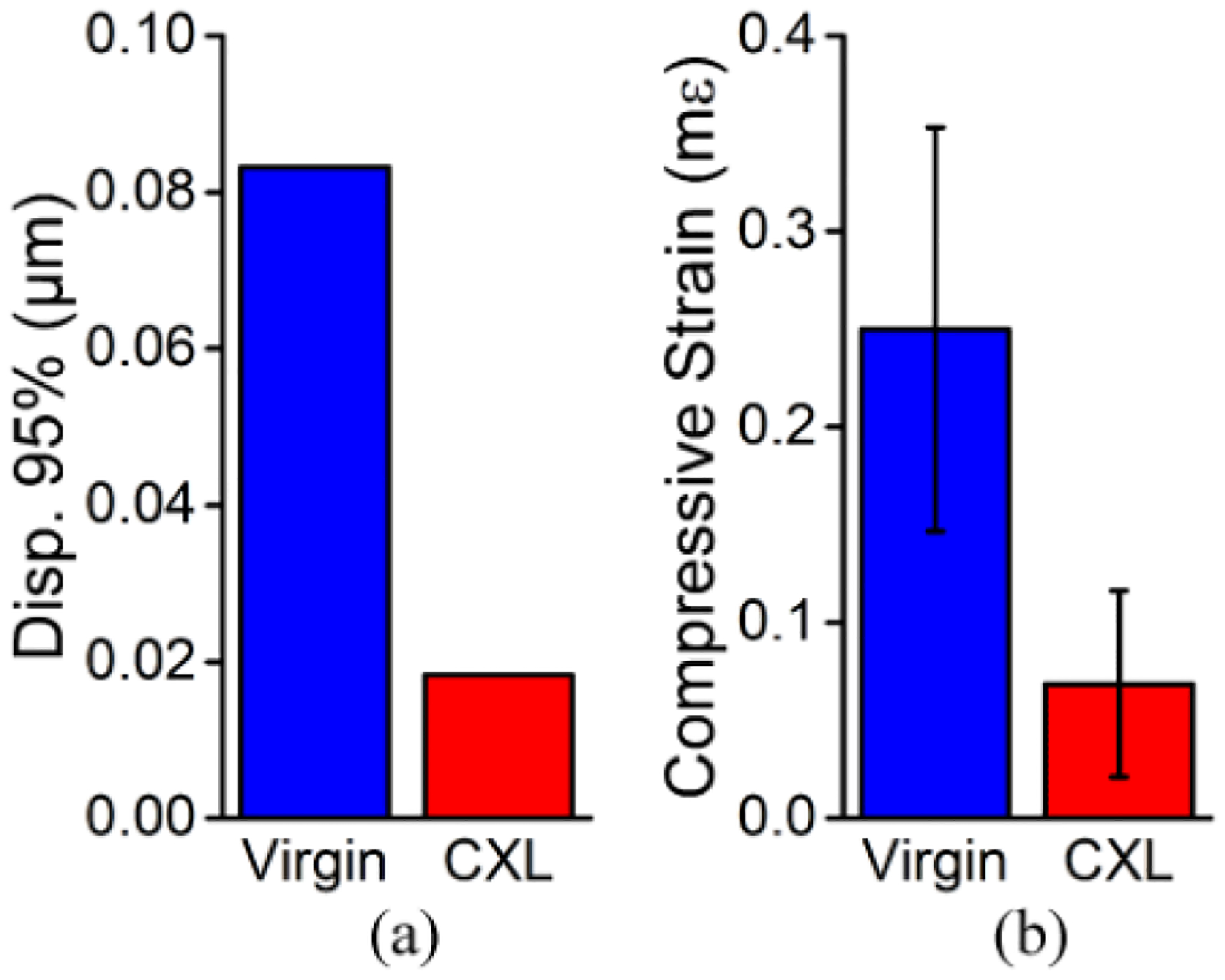
Quantitative analysis of the full-CXL in vivo rabbit cornea (N = 1). (**a**) 95th percentile of the displacement and (**b**) average compressive strain before and after CXL. Data are presented as the intra-sample mean ± standard deviation in (**b**).

The quantitative results from the in vivo cornea are plotted in Figure 9. The 95th percentiles of the displacement in the in vivo cornea before and after CXL were 0.08 μm and 0.02 μm, respectively. The compressive strain in the cornea before CXL was 0.25 ± 0.10 mε, which decreased by 75.2% to 0.07 ± 0.05 mε after CXL and was significant by a paired *t*-test (P < 0.001), indicating a drastic increase in corneal stiffness. The IOP of the rabbit eye was measured with a rebound tonometer (ICare TONOVET, ICare Finland Oy, Vantaa, Finland) before and after CXL, with a total of 5 measurements in each case. There was no significant change in the IOP (P = 0.0625) after CXL (virgin: 15.0 ± 0.0 mmHg, CXL: 16.8 ± 0.4 mmHg).

**Figure 10.**
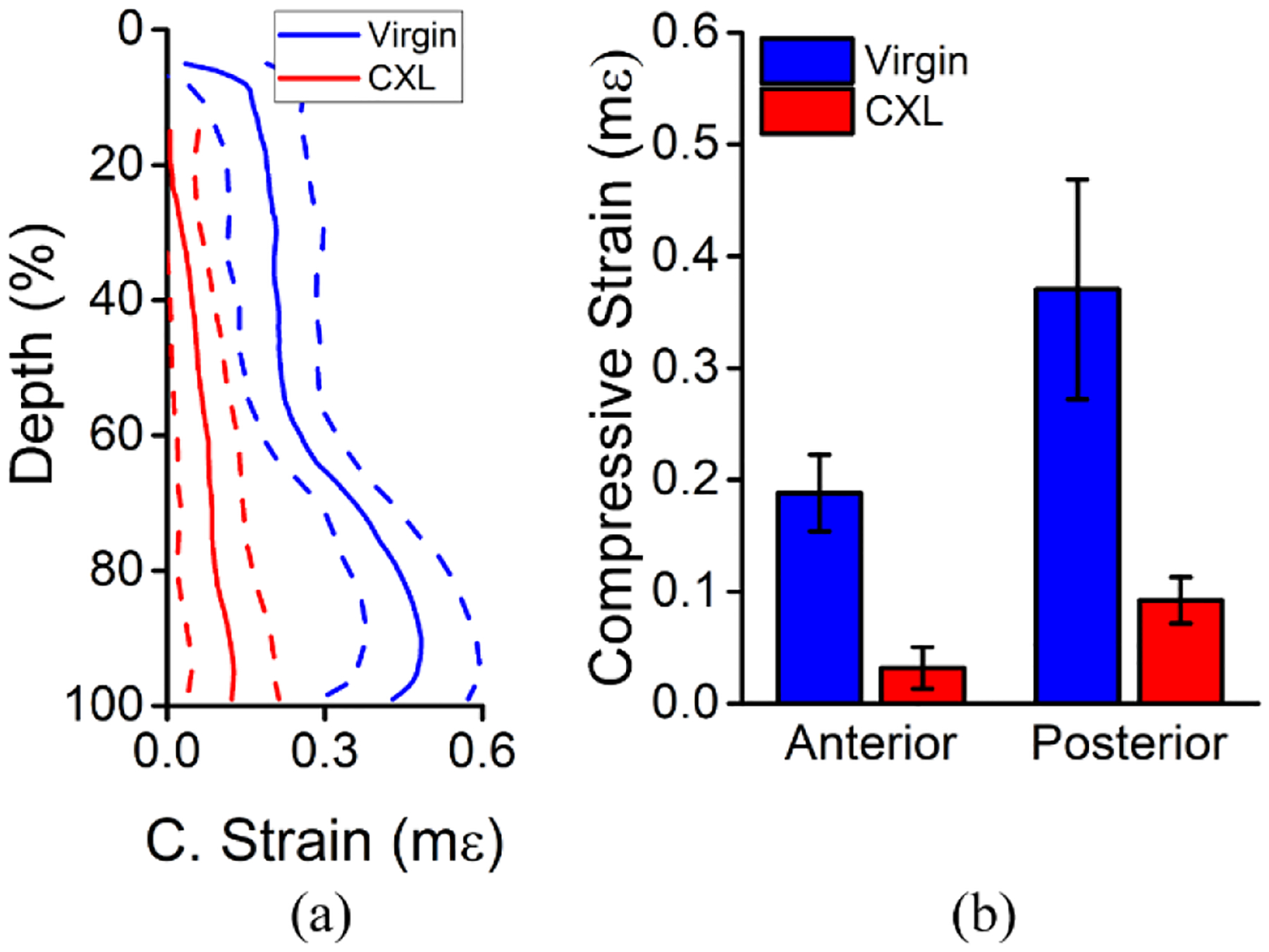
Depth-wise analysis of the full-CXL in vivo rabbit cornea (N = 1). (**a**) Laterally averaged and (**b**) regional analysis of the compressive strain before and after CXL. The solid lines in (**a**) are the mean, and the dashed lines are one standard deviation of error. The data in (**b**) is the intra-region mean ± standard deviation.

The depth-wise analysis of the in vivo rabbit cornea is plotted in Figure 10. The compressive strain was averaged laterally and shows a clear decrease in compressive strain (i.e., stiffening) of the cornea after CXL in Figure 10a. Regions near the interface between the compression plate and the cornea were removed due to large variations in displacement. The regional analysis showed a very significant difference in the compressive strain of the anterior and posterior regions by a one-tailed paired *t*-test (P < 0.001). Similarly, there was a very significant reduction in the compressive strain of the anterior (P < 0.001) and posterior (P < 0.001) regions after CXL, as plotted in Figure 10b.

**Figure 11.**
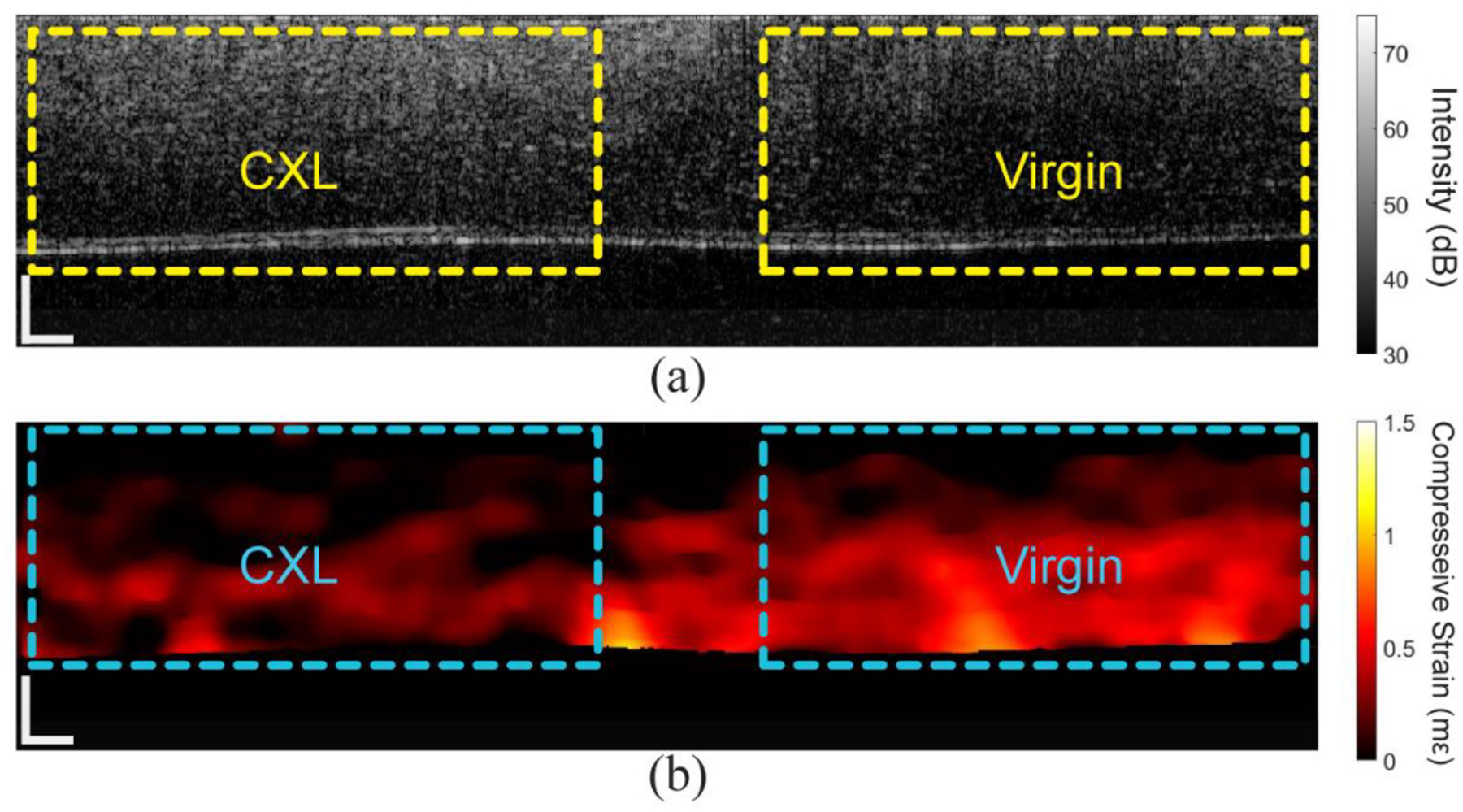
OCT and OCE images of the half-CXL in vivo rabbit cornea. (**a**) OCT image and (**b**) compressive strain map. The dashed boxes show the regions utilized for quantitative analyses. The scale bars are 100 μm.

**Figure 12.**
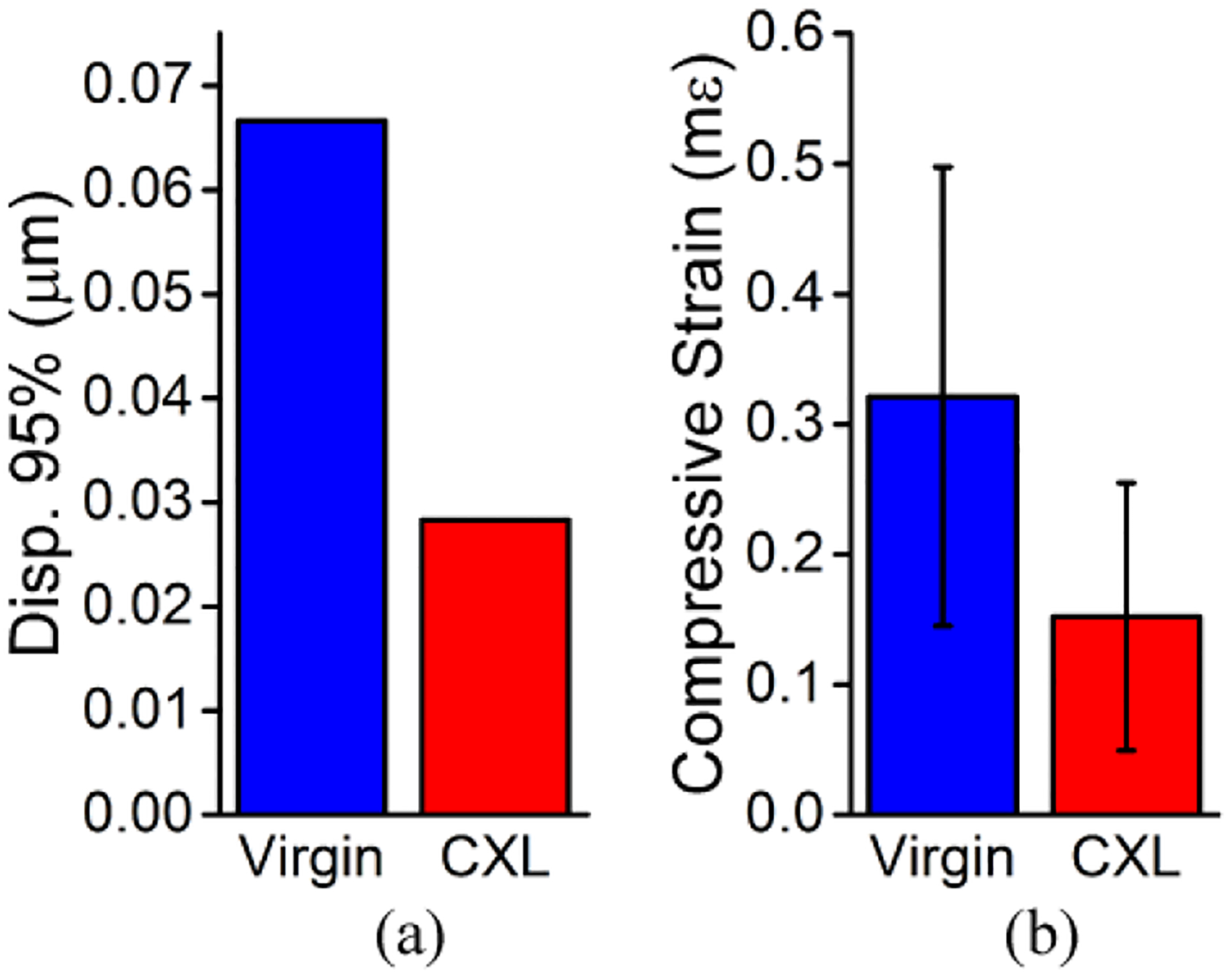
Quantitative analysis of the half-CXL in vivo rabbit cornea (N = 1). (**a**) 95th percentile of the displacement and (**b**) average compressive strain in the dashed regions in Figure 8. Data are presented as the intra-region mean ± standard deviation in (**b**).

In addition to the traditional CXL technique, partial CXL was performed on an additional cornea in another animal. The OCT image and compressive strain map are shown in Figure 11. There is a clear difference in scattering in the OCT image and the strain in the OCE image between the two regions, but the difference in thickness is not quite as clear as the in situ case in Figure 5. This is most likely due to the functioning endothelial cells that help maintain hydration. The quantitative results are plotted in Figure 12. The 95th percentile of displacement in the virgin region was 0.07 μm, but only 0.03 μm in the CXL region. The average compressive strain in the virgin region was 0.32 ± 0.17 mε, which was significantly greater (P < 0.001 by a one-tailed paired *t*-test) than the average compressive strain in the CXL region of 0.15 ± 0.10 mε, which corresponded to a decrease of ~52%.

**Figure 13.**
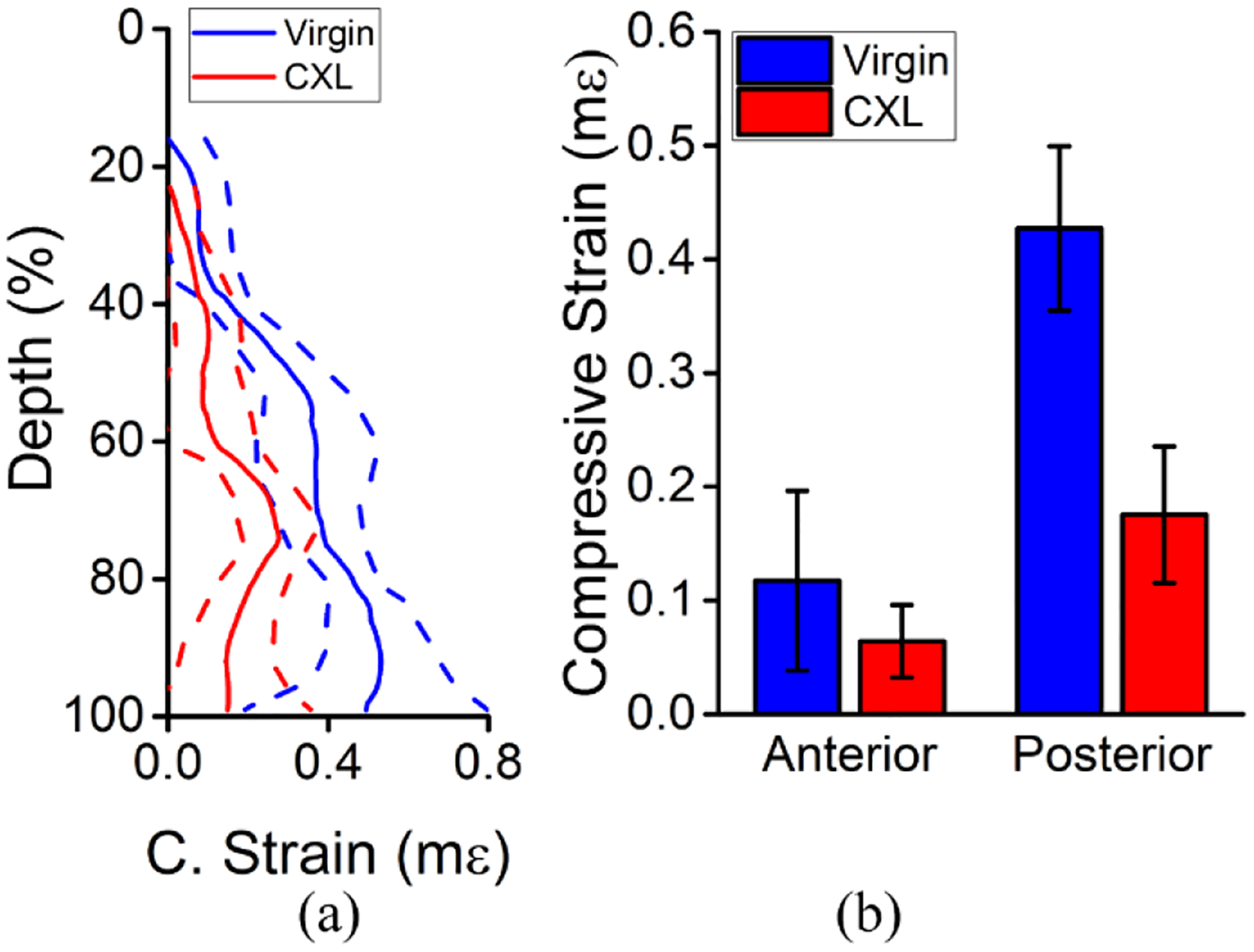
Depth-wise analysis of the half-CXL in vivo rabbit cornea (N = 1). (**a**) Laterally averaged and (**b**) regional analysis of the compressive strain of the virgin and CXL parts of the cornea. The solid lines in (**a**) are the mean, and the dashed lines are one standard deviation of error. The data in (**b**) is the intra-region mean ± standard deviation.

Figure 13 shows the results of the depth-wise regional analysis for the half-CXL in vivo sample. There was a very significant difference as tested by a one-tailed paired *t*-test between the compressive strain in the anterior and posterior regions of the cornea (P < 0.001). Similarly, there was a very significant difference between the virgin and CXL parts of the anterior region of the cornea (P < 0.001), which was the same for the posterior region of the cornea (P < 0.001) as tested by a one-tailed *t*-test.

## 4. Discussion

In this work, we demonstrated a compression-based optical coherence elastography technique for mapping the stiffness of the cornea under various conditions, including in situ and in vivo, and before and after various treatments designed to change corneal stiffness, including traditional CXL and partial CXL. The results show a significant increase in stiffness of the cornea as a function of the IOP and after CXL, which was consistent across 4 different cases (whole CXL in situ, partial CXL in situ, whole CXL in vivo, and partial CXL in vivo). These results emphasize the reliability of compression based OCE to measure stiffness changes in the cornea. More importantly, the addition of the stress measurements did not provide any significant change in the ability of compression OCE to detect changes in corneal stiffness induced by CXL. However, the change in compressive strain was not significantly affected by the baseline IOP, but when stress was accounted for, the baseline IOP did have a significant effect on the corneal pseudo-elasticity as quantified by Equation (2).

The in situ results showed a significant effect of the baseline IOP on the ΔIOP between the unloaded and loaded states. This is intuitive as the cornea exhibits non-linear biomechanical properties (i.e., its stress-strain curve is not linear) [39]. Hence, as the baseline IOP was increased, the stiffness of the cornea increased, and more force was transmitted through the cornea to the aqueous humor, resulting in a greater change in IOP between the loaded and unloaded states and decreased displacement and strain. This stiffening as a function of the IOP is well-noted in the literature and has been demonstrated repeatedly by various techniques, most notably elastography [40,41]. However, CXL did not have a significant effect on the ΔIOP, which may be due to the interplay between the change in corneal thickness and the increase in corneal stiffness [42,43]. For example, a change in the thickness of the cornea by dehydration can cause a dramatic increase in its stiffness when controlling for other parameters such as IOP [42], but only when accounting for the change in thickness. Similarly, the 95th percentile of the displacement was not significantly affected by the baseline IOP nor the CXL treatment. This may be likely due to the same reason, which is why the strain is a more indicative marker of stiffness. However, the compressive strain showed a much clearer difference between the virgin and CXL corneas. This can be explained, again, by the change in the thickness; although the displacements were relatively similar, the differences in thickness resulted in more clear results in the compressive strain. Nevertheless, the displacements were still quantified to demonstrate that the displacement is not always indicative of stiffness. On the other hand, there was an almost monotonic increase in corneal stiffness (pseudo-elasticity) as quantified by Equation (2) as a function of baseline IOP up until 30 mmHg once the stress was accounted for, and the results show an ~85% increase in stiffness after CXL.

In the half-CXL in situ cornea, statistical testing for the data as a function of the baseline IOP was not performed due to the small number of samples per baseline IOP value (1 data point per baseline IOP value). Nevertheless, there was a very clear trend in the ΔIOP as a function of the baseline IOP, similar to the virgin and normally treated CXL corneas. The 95th percentile of the displacements was significantly smaller in the CXL region as compared to the corresponding virgin regions. The displacements also had a clear trend as a function of the baseline IOP. This contrasts with the fully-CXL samples, where the displacements did not have a clear trend, which may have been due to the lateral distribution of forces between the two different regions in the half-CXL sample, which was not accounted for. There was also a trend in the strain, which slightly decreased as a function of the baseline IOP, indicating stiffening of the cornea. There was a clear trend in the stiffness as quantified by Equation (2) as a function of the baseline IOP that would not be fully captured by the Kruskal–Wallis ANOVA due to the small number of the samples. There was a significant difference between the virgin and CXL treated region stiffness quantified by Equation (2), demonstrating that the compression OCE technique could quantify and distinguish CXL-treated and untreated regions in the same cornea. This is particularly noteworthy because custom-CXL trials have shown that custom CXL procedures can have better outcomes with reduced side-effects [44], which could further be customized by biomechanical guidance [45].

The in vivo results showed clear differences in the displacement in both the full-CXL and half-CXL cases, which is in contrast to the in situ cases. As explained earlier, this is likely due to the change, or lack of change, in the thickness of the samples. Consequently, there were also significant differences in the compressive strain between the virgin and CXL corneas, indicating a clear stiffening effect of CXL that could be measured by compression OCE. These results emphasize the capability of compression OCE for measuring corneal stiffness in vivo. The in situ experiments were conducted in the whole eye globe configuration, and the eye globes were cannulated for artificial IOP control. This enabled simultaneous monitoring of the IOP, as shown in Figure 2. Clearly, the effects of loading and unloading are visible in the IOP measurements. These measurements were then further utilized to translate the qualitative strain in the cornea into a semi-quantitative measure of stiffness with Equation (2). In the live experiments, however, there was no cannulation, so measurement of the stress on the cornea was not possible. This can be easily overcome by utilizing a stress sensor [46], which is the next step of our work. Nevertheless, the results show the ability of compression-OCE to detect overall changes in corneal stiffness and measure spatial mechanical heterogeneity, which was induced by half-CXL. Thus, compression OCE may be able to detect localized corneal disease [2] and provide guidance for and evaluate custom procedures such as CXL, which have superior results compared to the current blanket treatment [44].

Applanating the cornea may cause discomfort in the clinic, but the use of a topical anesthetic can easily ameliorate any discomfort. For example, the gold standard of Goldmann applanation tonometry (GAT) is usually performed with a topical anesthetic, but Baptista et al. showed that GAT, even without any topical anesthetic, was well-tolerated [47]. De Stefano et al. have demonstrated clinical applications of a similar compression OCE technique [48]. By combining a force sensor attached to the compression plate with speckle tracking, they were able to detect a small but significant difference in the stiffness of keratoconic and normal corneas. The presented technique may be able to distinguish the biomechanical properties of corneal tissues with greater accuracy and discrimination due to much smaller displacements (μm scale versus almost 1 mm) and phase-sensitive detection, which would limit non-linear tissue responses, poroelastic effects, movement of the eye-globe, and very large changes in IOP. Moreover, a stress sensor could map stress heterogeneity [46], which may be crucial in the case of the curved cornea, localized diseases, and custom CXL. Another limitation of the proposed technique is the time needed for 3D imaging. In this work, only 2D imaging was performed, which took 1.6 s per OCE scan (for 10 pairs of B-scans). This could be drastically reduced with a greater incident power coupled with a faster imaging rate and fewer pairs of B-scans. Moreover, volumetric imaging at video-rate has been demonstrated by our group [49], and implementing such a technique for compression-based OCE of the cornea is the next step of our work. Another potential application of this technique is to utilize the ocular pulse to induce the stress in the cornea, as demonstrated by our recent work [30,50]. However, this relies on the heartbeat instead of the mechanical actuator, which would greatly increase imaging times. In comparison with dynamic wave-based techniques, static techniques have been clinically validated (e.g., Goldmann tonometry [51]), minimize extraneous motion due to contact with the compression plate [30,50], and faster imaging times, particularly for 3D imaging, due to B-M-mode imaging compared to M-B-mode imaging.

Direct comparison of the presented results with the literature is difficult due to differences in measurement type and scale. Previous phase-sensitive OCE techniques have been able to measure the stress-strain curve of the cornea with a compliant sensor [52]. However, these corneas were excised, so there was no control of IOP, and the strains measured in our work were orders of magnitude lower. Beyond compression-based OCE, Kling et al. found an ~58% increase in corneal stiffness after CXL utilizing numerical simulations based on inflation testing results [53], but Matteoli et al. found a ~41% increase utilizing inflation testing [54]. Seifert et al. utilized atomic force microscopy to characterize the regional stiffening of porcine corneas after CXL and found a range of increase in elasticity from 7.6× to 1.5× from the anterior to the middle of the cornea, respectively [55]. Strip extensiometry shows a wide range of results in the increase of porcine cornea stiffness after CXL as well. For example, the seminal publication on CXL stiffening showed a 1.8× increase [39]. Schumacer et al. showed an increase of ~1.3× [56], and Herbert et al. demonstrated an increase of ~1.7× [54]. Brillouin microscopy has shown that there is stiffening of the cornea for the anterior ~60% of the thickness [57]. However, Brillouin microscopy is not truly quantitative due to its measurement dependence on multiple parameters even when elasticity is controlled (e.g., hydration [13]). Similarly, wave-based elastography techniques show a great variation in results as well [40,41]. This may be due to various factors, such as hydration and storage media [42,58–61], age of the corneas [62,63], species [64], and general degradation of the ocular tissues after enucleation. Inter-technique quantitative measurements of corneal elasticity are not comparable either due to the scale of the tissue measurement (nanoscale with AFM to bulk measurements with inflation testing) and technique (such as Brillouin microscopy). We, however, can make comparisons in mechanical contrast, particularly for the half-CXL cases where direct comparison between the untreated and CXL regions is relatively straightforward. For example, in this work, our results show ~22% increase in the in situ case and a ~133% increase in the in vivo case, which is less than our previous work where the CXL regions were ~170% stiffer than the untreated region of an in situ porcine cornea at 15 mmHg. Although the mechanical contrast is limited in the compression technique, the wave-based technique estimation is solely based on the wave speed, which is not truly indicative of the stiffness [34,42]. These issues are further compounded by the anisotropic properties of the corneal tissue, both longitudinal and transverse. Pitre et al. have proposed a model of the cornea that accounts for the differences in corneal elasticity (i.e., tensile and shear) utilizing a wave-based OCE technique [65]. One of the future steps of our work is to combine the demonstrated compression-based approach with a transverse wave based OCE technique [66] and appropriate analytical model [34] to obtain a better insight into the whole picture of corneal biomechanical properties.

Our results showed a clear distinction in the stiffness of the anterior and posterior cornea, which has been confirmed by various methods [21,57,67–70], including compression OCE [23]. However, our results show stiffening of the anterior and posterior regions of the cornea after CXL, which contrasts with previous work that showed that only the anterior region is stiffened [68,69,71]. The results were not so clear in the in situ half-CXL case. This could be due to various factors, such as the assumption that the stress is uniform (laterally and axially), which is not strictly true, resulting in stiffness measurements with limited contrast [72]. Due to the common path imaging paradigm, the displacements at the anterior region of the cornea (i.e., proximal to the compression plate) will always be smaller than the displacements at the posterior region of the cornea (i.e., further away from the compression plate). This would potentially contribute to a greater degree of noise in the calculated strain in the anterior cornea because of the linear fitting process and contribution of noise to the resulting slope [38,73,74]. However, our results and results from others utilizing compression OCE show that this is not necessarily true, as shown by studies in homogeneous phantoms [38,71]. On the other hand, OCE measurements from deeper within samples show increased noise as compared to the anterior region due to the attenuation of the OCT signal. However, the corneas used in this work were sufficiently thin, and the focus of the OCT beam was placed in the middle of the samples so that the OCT signal contrast was relatively uniform through the entire corneas as seen from the structural images. Due to the relatively small amplitude of the displacements, particularly in the CXL corneas, there could be a relatively large contribution of noise to the linear fitting process [71,75]. Hence, large amplitudes would reduce the noise in the resulting elastograms and could enable measurements of stress-strain curves in the cornea [52]. Our future work is focused on finding the balance between sufficient displacement amplitude to produce high quality and accurate elastograms and minimal changes in IOP. Another possible source of error could be friction between the compression plate and the cornea [37,71]. When friction is present, there can be a noticeable non-linearity in the strain of a homogeneous sample, particularly near the compression plate. However, oil was utilized as a lubricant between the cornea and the compression plate to minimize this effect. The difference in thickness between the two regions could drastically affect the contact between the cornea and the compression plate because the natural shape of the cornea is curved. Development of more advanced mechanical models that account for the flattening of the cornea from its normal curved shape [74], including numerical simulations, detection of 2D/3D displacements [73], and the combination a stress sensor on the anterior cornea [71] combined with the IOP measurements is the next step of our work.

## 5. Conclusions

This work demonstrated the feasibility and robustness of utilizing compression-based optical coherence elastography for measuring the biomechanical properties of the cornea. The results show that although the compressive strain may not increase, particularly in in situ or ex vivo measurements, accounting for the stress shows a clear indication of stiffening in the cornea as IOP was increased and after CXL was performed. Our results also show the ability of the presented technique to measure spatial mechanical heterogeneity laterally and axially, which is critical for high-resolution mapping of corneal biomechanical properties. Most importantly, our results show that compression based OCE had repeatable results across 4 different scenarios in the cornea, so compression based OCE may be useful for high-resolution and quantitative mapping of corneal properties in clinical applications.

## Data Availability

Data are available from the authors on request.
